# Emotional and cognitive influences on alcohol consumption in middle-aged and elderly Tanzanians: a population-based study

**DOI:** 10.1038/s41598-024-64694-1

**Published:** 2024-07-30

**Authors:** Shuyan Liu, Patrick Kazonda, Germana H. Leyna, Julia K. Rohr, Wafaie W. Fawzi, Sachin Shinde, Ajibola Ibraheem Abioye, Joel M. Francis, Charlotte Probst, David Sando, Mary Mwanyka-Sando, Japhet Killewo, Till Bärnighausen

**Affiliations:** 1 Department of Psychiatry and Psychotherapy (Campus Charité Mitte), Charité – Universitätsmedizin Berlin, Berlin, Germany; 2 German Center for Mental Health (DZPG), Berlin and Heidelberg, Germany; 3 Dar Es Salaam Urban Cohort Study, Dar Es Salaam, Tanzania; 4 Department of Epidemiology, Muhimbili University of Health and Allied Sciences, Dar Es Salaam, Tanzania; 5 Department of Global Health and Population, Harvard T.H. Chan School of Public Health, Boston, Massachusetts USA; 6 Department of Family Medicine and Primary Care, Faculty of Health Sciences, University of the Witwatersrand, Johannesburg, South Africa; 7 Heidelberg Institute of Global Health (HIGH), Heidelberg University, Heidelberg, Germany; 8 Institute for Mental Health Policy Research, Centre for Addiction and Mental Health, Toronto, ON Canada; 9 Department of Psychiatry, University of Toronto, Toronto, ON Canada; 10 Management and Development for Health, Dar Es Salaam, Tanzania; 11 Africa Academy for Public Health, Dar Es Salaam, Tanzania; 12 Africa Health Research Institute, Somkhele and Durban, South Africa

**Keywords:** Mental health, Depressive symptoms, Cognitive performance, Drinking behaviour, Sub-Saharan Africa, Zero-inflated negative binomial regression model, Risk factors, Epidemiology

## Abstract

Alcohol consumption in Tanzania exceeds the global average. While sociodemographic difference in alcohol consumption in Tanzania have been studied, the relationship between psycho-cognitive phenomena and alcohol consumption has garnered little attention. Our study examines how depressive symptoms and cognitive performance affect alcohol consumption, considering sociodemographic variations. We interviewed 2299 Tanzanian adults, with an average age of 53 years, to assess their alcohol consumption, depressive symptoms, cognitive performance, and sociodemographic characteristics using a zero-inflated negative binomial regression model. The logistic portion of our model revealed that the likelihood alcohol consumption increased by 8.4% (95% confidence interval [CI] 3.6%, 13.1%, *p* < 0.001) as depressive symptom severity increased. Conversely, the count portion of the model indicated that with each one-unit increase in the severity of depressive symptoms, the estimated number of drinks decreased by 2.3% (95% CI [0.4%, 4.0%], *p* = .016). Additionally, the number of drinks consumed decreased by 4.7% (95% CI [1.2%, 8.1%], *p* = .010) for each increased cognitive score. Men exhibited higher alcohol consumption than women, and Christians tended to consume more than Muslims. These findings suggest that middle-aged and elderly adults in Tanzania tend to consume alcohol when they feel depressed but moderate their drinking habits by leveraging their cognitive abilities.

## Introduction

Alcohol consumption has raised major public health and socio-economic concerns worldwide. In Tanzania, 30% of patients seeking acute injury care tested positive for alcohol use^1^, with problematic drinking associated with a six-fold increase in the odds of injury^2^. Moreover, there is a dose-dependent relationship between the amount of alcohol intake and the odds of injury^1^. Apart from the health risks associated with problematic alcohol use^3^, its broader societal impacts extend to healthcare costs, the risk of infectious diseases, crime and antisocial behaviours^4^.

Moreover, alcohol use among middle-aged and older adults is increasingly common and has been trending upward over the years^5,6^. A national epidemiologic survey on alcohol in the United States showed substantial increases in alcohol use (22%), high-risk drinking (65%), and alcohol use disorder (107%) among older adults between 2001 and 2013^7^. Such a longitudinal survey in Sub-Saharan Africa – currently lacking – is highly desirable. Unlike younger adults, middle-aged and older adults are at greater risk to problematic alcohol use due to age-associated psychophysical and neurophysiological degenerations^6,8^. However, alcohol-related problems in middle-aged and older adults often remain hidden. The signs of problematic alcohol use can mimic geriatric syndromes and be masked by comorbid physical or psychiatric illness, making detection challenging^9^. Meanwhile, the global population is rapidly ageing. By 2050, the World Health Organization estimates that there will be 2 billion people aged over 60 (22% of the world population), with 80% of older people living in low- and middle-income countries (https://www.who.int/news-room/fact-sheets/detail/ageing-and-health). Rising rates of alcohol use among middle-aged and older adults, combined with alcohol-related multimorbidity, may bring new challenges to already burdened healthcare systems^10^. There is a lack of knowledge about alcohol use among middle-aged and older adults in low- and middle-income countries^11^.

In addition, cultural, socioeconomic, and environmental contexts can profoundly shape alcohol consumption patterns and subsequent healthcare outcomes^12–14^. These factors span a broad range, including religious beliefs, gender norms, economic conditions, the impact of the pandemic, and healthcare policies and financing^15–21^. For instance, in Islamic cultures, where alcohol consumption is restricted, intake rates are generally lower, resulting in fewer alcohol-related health issues^15,16^. Similarly, in certain contexts, societal norms discourage women from drinking, further affecting alcohol intake^17^. Other factors such as poverty, unemployment, and income status can contribute to variations in alcohol consumption patterns^18,19^. Furthermore, the pandemic^22^ can introduce new dynamics, affecting alcohol consumption through changes in availability, accessibility, and social restrictions^20,23^. To address alcohol-related health behaviours and outcomes, it is essential to implement effective detection and diagnosis techniques^24^, improve healthcare financing and regulation^21,25–27^, as well as enhance health education and awareness^28,29^. By examining context-specific factors, insights can be gained to develop tailored interventions and healthcare strategies to address alcohol-related health issues effectively.

Given the substantial burden of alcohol-related problems, research on alcohol remains critically lacking in Tanzania. Previous studies in Tanzania have primarily focused on the sociodemographic factors associated with alcohol consumption^30–33^. For instance, heavy alcohol use is more prevalent among men and non-Muslims than women and Muslim^30,31^. However, there remains a gap in understanding the psychological processes that underlie problematic drinking, particularly the role of cognitive performance in regulating the negative emotions that drive alcohol consumption^34,35^. It is well known that people often drink alcohol to cope with negative emotions^36^. They may consume alcohol to alleviate unpleasant emotional states, benefiting from its acute anxiolytic effects^37,38^. However, over time, this maladaptive behaviour become less responsive to cognitive ability, reinforcing the relationship between negative emotions and compulsive alcohol use^39^. Cognitive performance can regulate this motivational process to keep drinking in check^40^. As such, the balance between negative emotions and cognitive performance may significantly determine drinking behaviour^40^. Despite its importance, the relationship between psycho-cognitive phenomena and alcohol consumption has garnered little attention in Tanzania to date.

One challenge in modelling alcohol consumption outcomes is appropriately accounting for the distribution of drinking patterns. These distributions often have a high frequency of zeros, representing non-drinkers and abstinent individuals, and a long right tail, representing heavy drinkers^41^. A common strategy is to reduce the information in the data to a dichotomous outcome (comparing zero versus non-zero) or to use log transformations^42^. However, zeros cannot be log-transformed, and other approaches, such as adding “1” to the count outcomes with an excess of zeros before log transformation, do not adequately solve the problem. When dealing with datasets featuring a considerable number of zero counts and overdispersion, it is essential to employ statistical methods capable of accommodating this phenomenon. In this study, we propose employing a statistical approach to uncover patterns of alcohol consumption exhibiting significant variability across multiple conditions (e.g., age gender, education level, and religion). Specifically, we advocate for the adoption of a zero-inflated negative binomial (ZINB) regression model^43^ for data analysis. This model effectively addresses the challenge posed by excess zeros and overdispersion frequently encountered in datasets with a large number of zero-count observations, such as when modelling the number of alcohol drinks consumed per week in the general population. In such cases, where the variance exceeds the mean, the assumption of the Poisson distribution is violated^44,45^. The ZINB distribution, a mixture model combining a negative binomial distribution (represented by a count component) and a logit distribution (represented by a zero component), is instrumental in this regard. The dependent variable can take nonnegative integer values, including 0, 1, 2, 3, and beyond. ZINB regression, therefore, can effectively capture the intricate distribution of the data and furnish a comprehensive understanding of the variables influencing alcohol consumption, even amidst datasets marked by intricate zero-counts patterns.

In our study, we aim to explore the relationship between psycho-cognitive phenomena and alcohol consumption among middle-aged and older adults in Tanzania, considering sociodemographic characteristics. We hypothesize that greater severity of depressive symptoms and lower cognitive performance will be associated with both an increased likelihood of alcohol consumption and a higher number of alcohol drinks. Furthermore, we expect that these effects will vary across different sociodemographic factors, such as education, gender, and religion.

## Methods

### Participants and procedure

The study was conducted between June 2017 and July 2018 in Tanzania, using home interviews as part of the “Health and Ageing in Africa: A Longitudinal Study of an INDEPTH Community (HAALSI)” project among older adults in Dar es Salaam^46,47^. The HAALSI sample was embedded within the Dar es Salaam Urban Cohort Study (DUCS), which operated as a health and demographic surveillance system (HDSS)^48^. Participants were recruited from residents and households in the Ukonga and Gongo la Mboto wards of Ilala district in Dar es Salaam, Tanzania. Field workers conducted in-person interviews at each participant’s home. The study was approved by the Ethics Committee of the Harvard T.H. Chan School of Public Health, Office of Human Research Administration (ref. C13–1608–02) and the Muhimbili University of Health and Allied Sciences. All procedures followed were in accordance with the Helsinki Declaration of 1975 and the ethical standards of the responsible ethics committee. Written informed consent was obtained from all participants prior to their inclusion in the study.

### Measurements

The interview included assessments of sociodemographic characteristics, such as age, gender, education level, and religion, as well as evaluations of alcohol consumption, depressive symptoms, and cognitive performance. To evaluate alcohol consumption, we employed the Daily Drinking Questionnaire (DDQ)^49^. Participants were asked about the frequency of their alcohol consumption (i.e., number of drinking days per week) and the quantity consumed (i.e., number of standard drinks consumed on drinking days per week) over the past month. Show-cards for standard drinks and a table of equivalent alcohol units per drink (beer, wine, liquor, and spirits) were provided as reference. Total alcohol consumption was calculated by multiplying the quantity by the frequency.

To measure the severity of depressive symptoms, we employed the 10-item Centre for Epidemiological Studies Depression Scale (CES-D-10)^50^. Each item had a Likert scale ranging from 0 (“rarely or none of the time”) to 3 (“all of the time”). Higher scores indicated greater severity of depressive symptoms. To estimate reliability, we calculated the Cronbach’s alpha value within our sample^51^.

To assess cognitive function, we employed the Oxford Cognize Screen (OCS-Plus)^52^. This tool prioritizes visual-oriented tasks, thus minimizing language requirements and cultural biases. It has been validated in low-literacy and socioeconomic settings, as well as among individuals characterized by healthy aging^53^. To assess reliability within our sample, we computed the Cronbach’s alpha value. We administered an adapted version of the HAALSI survey in South Africa^54^, which included tasks for immediate and delayed recall, counting, numeracy, and orientation^55^. The overall score ranged from 0 to 26 points, with higher scores indicating better cognitive performance. In the immediate and delayed recall tasks, participants were asked to memorize a list of 10 words and then recall as many words as possible immediately and again after a timed delay interval (1 point for each correctly recalled word, totalling 20 points for both immediate and delayed recall). In the numeracy task, participants were required to complete the numeric sequence starting with two, four, and six (1 point). In the counting task, participants were asked to count sequentially from one to twenty (1 point). In the orientation task, participants were asked to answer the current year, month, date, and president of Tanzania (1 point for each correct answer, totalling 4 points).

### Data analysis

We conducted statistical analyses using *R version 4.1.0.* (www.r-project.org). To handle observations with missing data in all variables, we opted not to remove them, as this could introduce bias into the model. Instead, we used random forest imputation to develop an unbiased estimate for missing values^56^. Specifically, we employed the ‘missForest’ function in *R* package^57^, which uses a random forest trained on observed values of a data matrix to predict the missing values. For variables with excessive zeros (i.e., individuals reporting zero drinks per week), we built a ZINB regression model^43^. This model simultaneously estimates logistic and count portions. We used the alcohol consumption (i.e., standard drinks per week) as the dependent variable, while CES-D-10 depression symptom scores and cognitive performance scores were treated as independent variables, controlling for gender (men versus women; coding: 0 versus 1), age (continuous variable), education level (catalogue variable), and religion (Christians versus Muslims; coding: 0 versus 1) in our analyses. The logistic portion reports odds ratios (OR) of excess zeros (i.e., the likelihood of reporting zero drinks), while the count portion describes incident risk ratios (IRR) for the number of drinks consumed. Due to the logistic link functions used in the model‐fitting procedure, we exponentiated coefficients in the model output to report the ORs for drinking alcohol compared to not drinking alcohol and IRRs for the number of drinks consumed. Differences were considered as statistically significant at *p* < 0.05 and highly statistically significant at *p* < 0.01.

## Results

### Descriptive characteristics

A total of 2299 adults (1429 women; age range: 32–103, Median = 50, Mean = 52.92, SD = 10.68) were interviewed at home in Tanzania. The sample characteristics are shown in Table 1. On average, participants reported consuming 2.51 standard drinks per week (SD = 8.53). Among all seven variables of interest (i.e., age, gender, educational level, religion, CES-D-10 depression symptom score, and cognitive score), 6.12% of missing values (985 out of 16,093) were imputed using random forest.

**Table 1:** Sample characteristics.

Variable	Sample (N = 2299)
Age, mean (SD)^a^	52.92 (10.68)
Sex, n (%)	
Men	717 (31.19%)
Women	1429 (62.16%)
Missing	153 (6.65%)
Education level, n (%)	
No schooling	357 (15.53%)
Nursery school	1 (0.04%)
Primary grade 1	8 (0.35%)
Primary grade 2	24 (1.04%)
Primary grade 3	26 (1.13%)
Primary grade 4	103 (4.48%)
Primary grade 5	17 (0.74%)
Primary grade 6	25 (1.09%)
Primary grade 7	1190 (51.76%)
Secondary grade 1	50 (2.17%)
Secondary grade 2	46 (2.00%)
Secondary grade 3	15 (0.65%)
Secondary grade 4	271 (11.79%)
Secondary grade 5	1 (0.04%)
Secondary grade 6	26 (1.13%)
Vocational training	35 (1.52%)
Partial University	4 (0.17%)
University	56 (2.44%)
Missing	44 (1.91%)
Religion, %	
Christian	1035 (62.16%)
Muslims	1224 (37.84%)
None/others	40 (1.74%)
Depressive symptom severity score (CES-D-10)^b^, mean (SD)	8.56 (6.02)
Cognitive performance score, mean (SD)	13.2 (3.33)
Number of standard drinks per week, mean (SD)	2.51 (8.53)

^a^SD: standard deviation.

^b^CES-D-10: the 10-item Centre for Epidemiological Studies Depression Scale (CES-D-10).

In our study, the measurement scales demonstrated acceptable reliability within our sample, as indicated by Cronbach’s alpha values^51^. The Swahili version of the CES-D-10 exhibited strong reliability with a Cronbach’s alpha value of 0.84. Furthermore, the OCS-Plus displayed consistent reliability with a Cronbach’s alpha of 0.62. A Cronbach’s alpha value ranging from 0.6 to 0.8 is considered acceptable^58^.

### Association between independent correlates and alcohol consumption

On one hand, the logistic portion of our zero‐inflated negative binomial model analysis revealed that participants exhibiting depressive symptoms were estimated to be 8.4% (95% confidence interval [CI]: 3.6%, 13.1%, *p* < 0.001) less likely to report zero drinks, indicating an increased likelihood of alcohol consumption as depressive symptom severity increased, shown in Table 2. However, cognitive performance scores showed no significant relationship with alcohol intake (*p* = 0.868).

**Table 2:** Zero-inflated negative binomial regressions predicting alcohol use (N = 2299).

Variables	OR/IRR	95% CI for IRR/OR		*P* value
		Lower	Upper	
Logistic portion of model (for predicting excess zeros, ***OR***)				
CES-D-10 score	0.916	0.870	0.964	< .001***
Cognitive score	1.005	0.951	1.060	.868
Age	0.992	0.974	1.010	.384
Education Level	0.934	0.886	0.985	**.012**
Gender (Men versus Women)	2.296	1.642	3.211	< .001***
Religion (Christians versus Muslims)	2.256	1.620	3.142	< .001***
Count portion of model (negative binomial regression with log link, ***IRR***)				
CES-D-10 score	0.978	0.960	0.996	**.016**
Cognitive score	0.953	0.919	0.988	**.010**
Age	0.983	0.971	0.995	.007
Education Level	1.045	1.007	1.083	.018
Gender (Men versus Women)	0.474	0.375	0.599	< .001***
Religion (Christians versus Muslims)	0.643	0.517	0.799	< .001***

CES-D-10: 10-item Centre for Epidemiological Studies Depression Scale.

SE: Standard error. OR: Odds ratio. IRR: Incidence risk ratio.

Significant values are in bold.

On the other hand, in the count portion of the model, each additional point in depressive symptom severity was associated with a decrease of 2.3% (95% CI [0.4%, 4.0%], *p* = 0.016) in the estimated number of drinks per week. Conversely, for each increase in cognitive performance score, there was a decrease of 4.7% (95% CI [8.1%, 1.2%], *p* = 0.010) in the estimated number of drinks per week, shown in Table 2. These results suggest that while middle-aged and older adults are more likely to drink alcohol when experiencing depressive symptoms, they do not drink excessively by leveraging their cognitive abilities.

In addition, in both logistic and count portions of the model, we found that higher educational levels, male gender, and Christian religion were associated with higher alcohol consumption. Specifically, individuals with higher education levels were more likely to consume alcohol compared to those with lower education levels. Moreover, men were more likely to have higher alcohol consumption compared to women, and Christians were more likely to consume alcohol compared to Muslims (all *P*s < 0.05).

## Discussion

We investigated the relationship between psycho-cognitive phenomena and alcohol consumption in Tanzania considering sociodemographic factors. Consistent with our hypothesis built upon previous studies conducted outside Tanzania^59^, we discovered that middle-aged and older adults in Tanzania tend to consume alcohol when they feel depressed. Surprisingly, we found that they exhibit moderation in alcohol intake, with increased severity of depressive symptoms and higher cognitive performance associated with lower numbers of alcohol drinks. Moreover, our findings revealed associations between alcohol use and sociodemographic factors, as expected. Specifically, individuals with higher education levels were found to consume more alcohol compared to those with lower education levels. Men exhibited higher alcohol consumption than women, and Christians consumed more alcohol than Muslims.

Our study highlights the relationship between psycho-cognitive status and alcohol‐drinking patterns among middle-aged and older adults in Tanzania. We discovered that while these individuals are more likely to consume alcohol when experiencing depressive symptoms, they do not tend to engage in excessive drinking as the severity of these symptoms increases. This unexpected evidence of moderation in alcohol intakes sheds light on the complex interplay between mental health and alcohol use. It facilitates further consideration of the potential mediation of alcohol consumption in the association between common mental disorders and cardiovascular disease as well as all-cause mortality^60,61^. Moreover, our study contributes to the advancement of mental health and alcohol research in the region, offering valuable insights to inform strategies aimed at preventing problematic alcohol use. On one hand, our findings highlight the importance of perceived social support in mitigating alcohol use as a coping mechanism for depression^62,63^. On the other hand, structural interventions are needed to address problematical alcohol use in Tanzania^64,65^. For instance, previous research conducted in Tanzania has demonstrated the association between alcohol advertising and drinking behaviour^65^, prompting the implementation of health warning labels on all alcohol advertisements as a preventive measure^64^. These efforts reflect the ongoing commitment to combating alcohol-related issues and promoting public health in Tanzania.

Contrary to our hypothesis, cognitive performance did not appear to be a significant determinant of whether middle-aged and older adults chose to drink or not. However, consistent with our expectations, we found that higher cognitive performance was associated with a lower number of drinks consumed. Thes mixed results indicate that while cognitive performance may not directly drive the decision to drink, it does play a role in regulating excessive alcohol consumption, particularly in relation to problematic alcohol use. Our results align with findings from a cross-sectional study by Humphreys and co-workers^54^, which found that higher cognitive performance was associated with lower alcohol use among older adults in South Africa. Conversely, another direction of the relationship has been identified: older adults in India who consumed alcohol had a 30% higher likelihood of experiencing cognitive impairment^66^. Future studies should further investigate the directionality and causality of these relationships.

Our study suggested that middle-aged and older adults in Tanzania do not drink excessively when they feel depressed, possibly by leveraging their cognitive abilities. This implies that individuals who maintain cognitive function may indeed refrain from engaging in problematic alcohol use. Psychoeducation, cognitive reappraisal, skills training, and other behavioural strategies could potentially aid in enhancing cognitive ability and consequently reducing the likelihood of problematic alcohol consumption. It is worth noting that our sample predominantly comprised middle‐and older‐age adults, deliberately lacking representation from younger individuals, as our research is cantered on studying aging populations. A cross-sectional and longitudinal study conducted among a large Dutch student sample did not find a significant association between cognitive performance and alcohol consumption^67^. Given that the peak age range for problematic alcohol consumption in Tanzania falls between 25 and 34 years^33^, there is a clear need for further research including a broad age range, as age may have an impact on cognitive performance. In addition, future studies could include other types of cognitive instruments to assess cognitive control (e.g., response inhibition) and decision-making processes (i.e., risk taking) in alcohol intake^68^. This approach could provide deeper insights into the intricate relationship between cognitive functioning and alcohol consumption across different age groups.

Furthermore, our study added to the existing evidence indicating that men tend to consume more alcohol than women, and Christians exhibit higher alcohol consumption compared to Muslims in Tanzania^30,32,33^. However, the association of alcohol use with a range of sociodemographic factors warrants further research.

The study has several limitations that warrant emphasis. First, while our findings provide valuable insights into associations, they cannot establish causality. To determines causal inferences, longitudinal observational studies are needed. Second, our participants were recruited solely from Dar es Salaam, which introduces geographical bias and limits the generalizability of our results to other parts of Tanzania or East Africa. Future studies should consider cross-country comparisons^69–71^, especially within Africa, to provide a more comprehensive understanding of alcohol consumption among middle-aged and older adults. Third, our current study did not assess drinking motives, perceptions of alcohol use^72^, or the underlying causes of depressive symptoms. Incorporating these aspects into more detailed qualitative studies would generate a more comprehensive understanding of the relationship between psycho-cognitive phenomena and alcohol consumption in Tanzania.

Our research holds valuable implications for both the healthcare system and managerial practices. Understanding the relationship between depressive symptoms, cognitive performance and alcohol consumption among middle-aged and older adults in Tanzania can provide valuable insights for healthcare providers, informing the need for tailored interventions and support services. For the healthcare system, our findings may catalyse the development of tools to identify and safeguard individuals at risk of problematic alcohol use, particularly those experiencing depressive symptoms, male gender, and adhering to Christian beliefs. Furthermore, our study may illuminate healthcare financing by employing financial econometrics^73^ to account for mental health costs and services, as well as the social and economic costs associated with alcohol-related health outcomes^74–76^. In addition, incorporating high-tech health infrastructure into healthcare planning^77^ and leveraging digital tools^78^ can enhance health service accessibility, scalability, and sustainability, ensuring comprehensive health coverage. These proactive approaches can facilitate cost-effective prevention and early intervention, thereby alleviating the burden on healthcare resources associated with alcohol-related issues^21^. From a managerial standpoint, our research highlights the importance of considering cognitive awareness and health education in addressing alcohol-related challenges^29^. This insight could be integrated into training programs, media advocacy, alcohol control policies, and community outreach initiatives aimed at promoting responsible alcohol consumption^79^. Overall, our research has the potential to inform strategic decisions and interventions within both healthcare and managerial contexts, ultimately contributing to improved public health outcomes and enhanced quality of life for individuals.

## Conclusion

Our study reveals that middle-aged and older adults in Tanzania are inclined to consume alcohol when experiencing depressive symptoms, yet they moderate their drinking behaviour by leveraging cognitive abilities. Building upon our findings, future research could explore various avenues to enrich our understanding and inform targeted interventions. This includes conducting comparative and spatial–temporal analyses^73,77,80,81^ across diverse regions to uncover variations in alcohol consumption behaviours, thereby guiding context-specific interventions. Moreover, further investigation into the underlying psycho-cognitive mechanisms driving alcohol consumption behaviours can inform the design of targeted interventions and policies^79^. Additionally, future studies can focus on exploring associated healthcare costs, burden on healthcare resources, and potential interventions to mitigate negative health outcomes related to alcohol consumption in specific contexts^21^. By pursuing these avenues of research, we can advance our knowledge of alcohol consumption behaviours and inform comprehensive psycho-cognitive strategies to address alcohol-related challenges and improve public health in Tanzania and beyond.

## Data Availability

The datasets analysed during the current study are available from the corresponding author on reasonable request.
